# Stress Hyperglycemia Ratio and Triglyceride–Glucose Index for Predicting In-Hospital Mortality and Heart Failure in Acute Myocardial Infarction

**DOI:** 10.3390/healthcare14213698

**Published:** 2026-11-01

**Authors:** Perihan Varim, Betül Sarıbıyık Çakmak, Ahmet Can Çakmak, Emre Eynel, Taner Demirci, Ersan Tatli

**Affiliations:** 1 Department of Cardiology, Focus Heart and Arrhythmia Center, Sakarya 54100, Turkey; 2 Department of Cardiology, Medicine Faculty, Sakarya University, Sakarya 54100, Turkey; betul.saribiyik@gmail.com (B.S.Ç.); drahmetcancakmak@gmail.com (A.C.Ç.); emreeynel@gmail.com (E.E.); 3 Department of Endocrinology, Focus Heart and Arrhythmia Center, Sakarya 54100, Turkey; tnrdemirci@gmail.com

**Keywords:** stress hyperglycemia ratio (SHR), triglyceride–glucose index (TyG Index), acute myocardial infarction

## Abstract

**Highlights:**

**What are the main findings?**

- Both the triglyceride–glucose (TyG) index and stress hyperglycemia ratio (SHR) were associated with in-hospital heart failure and mortality in patients with acute myocardial infarction undergoing primary PCI.
- SHR showed better discriminative performance for in-hospital mortality than the TyG index and remained an independent predictor of mortality after multivariable adjustment.

**What are the implications of the main findings?**

- SHR, which integrates acute glucose elevation with baseline glycemic status, may provide a simple and readily available marker for early risk stratification in high-risk patients with acute myocardial infarction.
- Assessing acute stress-related hyperglycemia alongside chronic metabolic dysfunction may improve the characterization of short-term cardiometabolic risk during acute myocardial infarction.

**Abstract:**

Background: Metabolic dysregulation substantially contributes to adverse outcomes in acute myocardial infarction (AMI). While the triglyceride–glucose (TyG) index reflects chronic insulin resistance, the stress hyperglycemia ratio (SHR) represents acute stress-related glycemic perturbation. Methods: In this retrospective cohort study, 494 patients with STEMI who underwent primary PCI were included. SHR was calculated using admission blood glucose, whereas the TyG index was calculated using glucose and triglyceride levels obtained from routine morning blood samples, generally collected between 04:00 and 06:00 a.m. The primary outcomes were in-hospital mortality and congestive heart failure (CHF). Multivariable logistic regression and receiver operating characteristic (ROC) analyses were performed to evaluate independent associations and discriminative performance. Results: Patients who developed CHF or in-hospital mortality exhibited significantly higher SHR and TyG index levels compared with those without adverse outcomes (all *p* < 0.05). SHR demonstrated a moderate positive correlation with TyG index (r = 0.441, *p* < 0.001), supporting the interplay between chronic insulin resistance and acute metabolic stress during AMI. For in-hospital mortality, SHR had a numerically higher area under the ROC curve [AUC: 0.823 (95% CI: 0.727–0.920)] than TyG [AUC: 0.714 (95% CI: 0.605–0.824)]; a formal comparison of the AUCs was not performed. At a cut-off value of 1.45, SHR demonstrated 76.5% sensitivity and 80.0% specificity for in-hospital mortality. In the original multivariable model, the SHR group was associated with in-hospital mortality (OR: 9.61, 95% CI: 2.599–35.578; *p* = 0.001), although the small number of deaths and incomplete adjustment for established clinical prognostic factors limit interpretation. Conclusions: Among patients with STEMI, both chronic insulin resistance and acute stress-related hyperglycemia were associated with adverse in-hospital outcomes. However, SHR demonstrated superior discriminative capacity and independently predicted mortality, highlighting the dominant prognostic impact of acute metabolic stress during AMI. Integrated assessment of chronic and acute metabolic dysfunction may improve early risk stratification in high-risk myocardial infarction patients.

## 1. Introduction

Acute myocardial infarction (AMI) remains one of the leading causes of cardiovascular mortality and morbidity despite major advances in reperfusion strategies and contemporary pharmacological treatment [1,2,3]. Patients presenting with acute coronary syndromes, particularly those with multivessel coronary artery disease (MVCAD), are at increased risk of developing serious in-hospital complications including heart failure, malignant arrhythmias, cardiogenic shock, and death [4,5]. The extent of myocardial injury, the severity of coronary atherosclerotic burden, and associated hemodynamic and metabolic disturbances play critical roles in determining short-term prognosis. Therefore, early recognition of high-risk patients during hospitalization remains essential for optimizing treatment strategies, improving clinical monitoring, and reducing adverse cardiovascular outcomes [6].

Insulin resistance and stress-related metabolic dysregulation have emerged as pivotal mechanisms linking cardiometabolic dysfunction to the progression of atherosclerotic disease [7,8]. Chronic impairment of insulin signaling contributes to endothelial dysfunction, oxidative injury, systemic inflammation, and accelerated plaque formation, thereby promoting both the development and progression of coronary artery disease (CAD) [9,10]. The triglyceride–glucose (TyG) index, derived from fasting triglyceride and fasting plasma glucose levels, has recently gained attention as a practical surrogate marker of insulin resistance. The TyG index offers several advantages, including wide availability, low cost, and ease of calculation in routine clinical practice. Accumulating evidence has demonstrated a significant association between elevated TyG index levels and coronary atherosclerotic burden, metabolic dysfunction and adverse cardiovascular prognosis in both diabetic and non-diabetic populations [11,12]. Furthermore, previous studies have shown that the TyG index may provide incremental prognostic information in patients with complex coronary artery disease, particularly those with multivessel involvement.

Acute stress hyperglycemia represents another important metabolic response associated with cardiovascular injury and unfavorable clinical outcomes [13]. Unlike isolated glucose measurements, the stress hyperglycemia ratio (SHR) incorporates both admission glucose and Hemoglobin A1c (HbA1c), thereby accounting for baseline glycemic status and more accurately reflecting relative stress-induced hyperglycemia. From a pathophysiological perspective, acute hyperglycemic stress may exacerbate myocardial injury through several mechanisms, including inflammatory activation, oxidative stress, endothelial dysfunction, platelet hyperreactivity, and microvascular impairment. These mechanisms may ultimately exacerbate myocardial injury and contribute to adverse cardiovascular outcomes. Recent investigations have demonstrated significant associations between elevated SHR levels and mortality, infarct severity, heart failure, and poor clinical outcomes in patients with acute coronary syndromes [14]. Nevertheless, the prognostic implications of SHR remain incompletely understood, as previous studies have reported inconsistent associations with long-term outcomes including linear J-shaped and U-shaped relationships.

Although the TyG index and SHR reflect distinct but potentially related aspects of metabolic dysfunction, evidence directly comparing their prognostic associations in patients with acute myocardial infarction remains limited [15]. Previous studies have generally evaluated these indices separately or in selected clinical populations, including patients with chronic total occlusion or acute coronary syndromes [16]. The associations of these metabolic indices with short-term clinical outcomes, particularly in patients with multivessel coronary artery disease, require further investigation. Therefore, the primary objective of this study was to evaluate the associations of the TyG index and SHR with in-hospital mortality and congestive heart failure in patients with STEMI undergoing primary PCI. The secondary objective was to assess their respective discriminative performance for in-hospital mortality.

## 2. Materials and Methods

### 2.1. Study Design and Population

This retrospective observational cohort study evaluated hospital records from the one-year period preceding ethics committee approval. The study protocol was approved by the Sakarya University Faculty of Medicine Ethics Committee on 8 February 2026 (Approval No. 43012747-050.04-572571-204). Retrospective screening and data extraction from electronic hospital records were conducted between April and June 2026, following ethics committee approval. The study was conducted in accordance with the principles of the Declaration of Helsinki.

All 494 patients included in the study presented with STEMI, underwent primary PCI with stent implantation in the culprit coronary lesion, and were subsequently admitted to the coronary intensive care unit. Our institution is a primary PCI-capable center where routine clinical practice aims to achieve a door-to-balloon time of less than 30 min.

STEMI was diagnosed according to the diagnostic criteria outlined in the current European Society of Cardiology (ESC) guidelines, based on clinical presentation, electrocardiographic findings, and cardiac biomarker levels.

Patients younger than 18 years and those with missing essential laboratory data, including fasting plasma glucose, triglyceride or HbA1c measurements were excluded. Additional exclusion criteria were severe valvular heart disease, congenital heart disease, active infection, chronic inflammatory disease, malignancy, advanced hepatic dysfunction, end-stage renal disease, thyroid disorders, a previous diagnosis of chronic heart failure, and incomplete clinical records.

### 2.2. Baseline Characteristics and Data Collection

Demographic characteristics, cardiovascular risk factors, medical history, clinical presentation, laboratory findings, electrocardiographic features, angiographic characteristics, and in-hospital outcomes were retrospectively obtained from electronic hospital records.

Hypertension and diabetes mellitus were defined based on documented medical history or the use of antihypertensive or antidiabetic medications. Smoking status was categorized as current smoker or non-smoker. Previous myocardial infarction and cerebrovascular disease were recorded based on previously physician documented diagnosis.

Myocardial infarction location was classified according to electrocardiographic findings as anterior, inferior, anteroseptal, anterolateral, inferoposterior, or right ventricular myocardial infarction.

### 2.3. Laboratory Measurements and Calculation of Metabolic Indices

Venous blood samples were analyzed using standard automated methods at the central biochemistry laboratory. Admission plasma glucose was measured from the blood sample obtained at hospital presentation and was used to calculate SHR. At our institution, routine morning blood samples are generally collected between 04:00 and 06:00, when glucose and lipid profiles are measured together. For TyG calculation, glucose and triglyceride measurements from the same morning sampling session were identified through retrospective review of patient records and used together. Thus, the glucose measurements used for SHR and TyG originated from separate sampling occasions. For patients presenting at night or in the early morning, the interval between these samples could be short. Other routine laboratory parameters, including HbA1c, were extracted from hospital records.

The triglyceride–glucose (TyG) index was calculated using the following established formula:

TyG = ln [Triglyceride (mg/dL) × Fasting plasma glucose (mg/dL)/2]

based on fasting laboratory values [17,18].

The stress hyperglycemia ratio (SHR) was calculated by dividing admission blood glucose by estimated average glucose derived from HbA1c using the following formula:

SHR = Admission blood glucose (mg/dL)/[28.7 × HbA1c (%) − 46.7]

Admission glucose was defined as the glucose measurement obtained from the blood sample collected at hospital presentation, rather than any measurement within the first 24 h [19].

All laboratory measurements were analyzed using standardized laboratory procedures by personnel blinded to the study outcomes.

### 2.4. Coronary Angiographic Evaluation

Coronary angiography was performed using standard techniques via radial or femoral arterial access according to current guideline recommendations. All angiographic procedures were performed by experienced interventional cardiologists. Angiographic images were independently evaluated by experienced interventional cardiologists who were blinded to patients’ clinical outcomes and metabolic index results.

Significant coronary artery disease was defined as ≥50% luminal stenosis in the left main coronary artery or ≥70% stenosis in the major epicardial coronary arteries, including the left anterior descending artery, left circumflex artery, and right coronary artery.

Multivessel coronary artery disease was defined as the presence of significant stenosis in at least two major epicardial coronary arteries on coronary angiography.

### 2.5. Echocardiographic Assessment

Transthoracic echocardiography was performed during hospitalization using commercially available ultrasound systems in accordance with current echocardiographic guidelines. Left ventricular ejection fraction (LVEF) was assessed using the modified Simpson biplane method.

All echocardiographic examinations were evaluated by experienced cardiologists blinded to patients’ clinical and laboratory data.

Congestive heart failure was defined as the development of clinical signs and symptoms consistent with heart failure during hospitalization, accompanied by a left ventricular ejection fraction (LVEF) <45% on transthoracic echocardiography.

### 2.6. Study Outcomes

The primary outcomes of the study were all-cause in-hospital mortality and the development of congestive heart failure during hospitalization.

Secondary analyses were performed to evaluate the predictive performance and prognostic value of the TyG index and SHR for adverse in-hospital clinical outcomes, particularly mortality risk stratification.

### 2.7. Statistical Analysis

Statistical analyses were performed using IBM SPSS Statistics for Windows, version 26.0 (IBM Corp., Armonk, NY, USA). The distribution of continuous variables was assessed using visual (histograms and probability plots) and analytical methods. Continuous variables were expressed as mean ± standard deviation or median (interquartile range) according to data distribution, while categorical variables were presented as numbers and percentages.

Comparisons between two independent groups were performed using the Mann–Whitney U test for non-normally distributed variables. Correlations between continuous variables were evaluated using Spearman correlation analysis.

Receiver operating characteristic (ROC) curve analysis was used to determine the predictive performance of SHR and TyG indices for mortality, and the area under the curve (AUC), sensitivity, specificity, and optimal cut-off values were calculated.

Univariate and multivariate logistic regression analyses were performed to determine independent predictors of in-hospital mortality. For logistic regression analyses, SHR and TyG were dichotomized using ROC-informed thresholds. Patients were classified into two groups according to SHR ≥ 1.45 versus <1.45 and TyG > 9.40 versus ≤9.40. These categorical variables were subsequently included in the logistic regression analyses to evaluate their associations with in-hospital mortality.

Variables with *p* < 0.10 in univariate analysis were included in the multivariate logistic regression model, and odds ratios with 95% confidence intervals were calculated. A *p* value < 0.05 was considered statistically significant.

## 3. Results

The study included 494 patients with STEMI, all of whom underwent primary PCI. Of these, 327 (66.2%) were male. Hypertension was the most common accompanying chronic disease (45.3%), and 5.9% of the patients had a previous history of myocardial infarction. During hospitalization, 17 patients (3.4%) died, while 70 patients (14.2%) developed congestive heart failure (Table 1). Laboratory findings are summarized in Table 2.

The distribution of myocardial infarction localization was as follows: inferior myocardial infarction in 212 patients (42.9%), anterior myocardial infarction in 211 patients (42.7%), anterolateral myocardial infarction in 30 patients (6.1%), inferoposterior myocardial infarction in 21 patients (4.3%), anteroseptal myocardial infarction in 11 patients (2.2%), and inferoposterior myocardial infarction with right ventricular involvement in 9 patients (1.8%).

**Figure 1:**
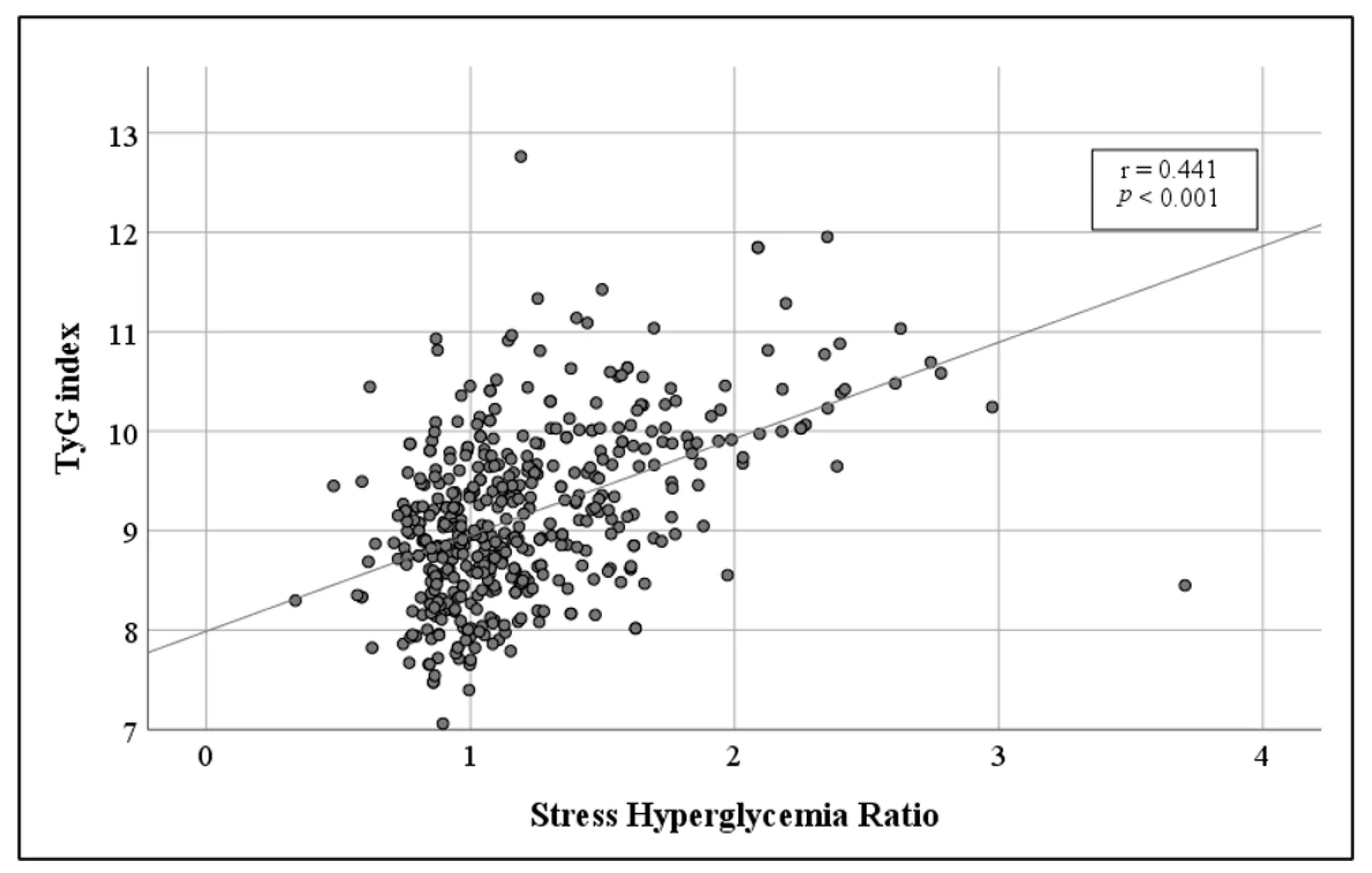
The correlation between the TyG (triglyceride-glucose) index and the stress hyperglycemia index (R^2^ linear: 0.227).

The relationship between the stress hyperglycemia ratio (SHR) index and the TyG index was evaluated using Spearman correlation analysis, which demonstrated a statistically significant positive correlation (r = 0.441, *p* < 0.001) (Figure 1).

**Figure 2:**
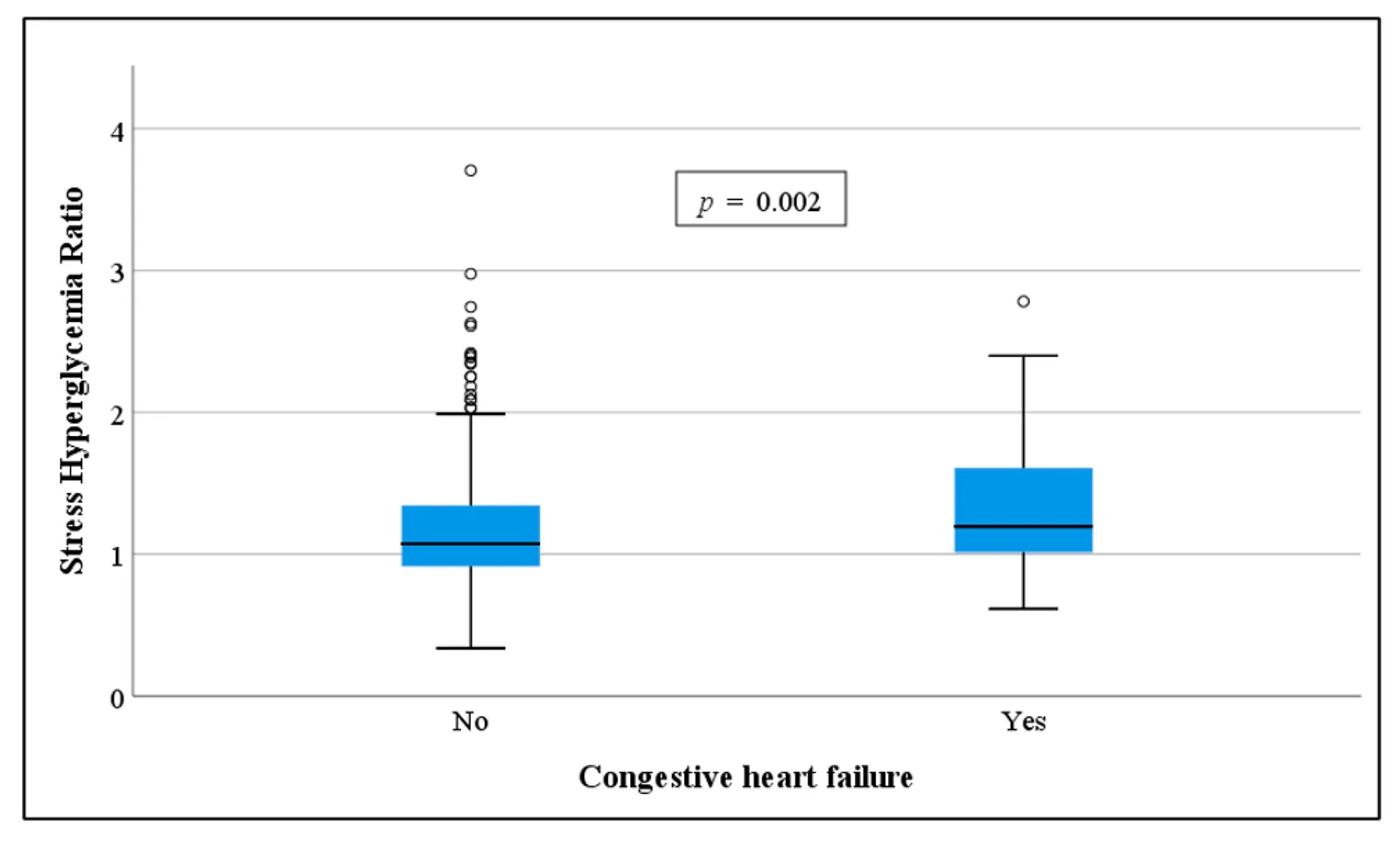
Comparison of SHR index according to the presence of congestive heart failure.

**Figure 3:**
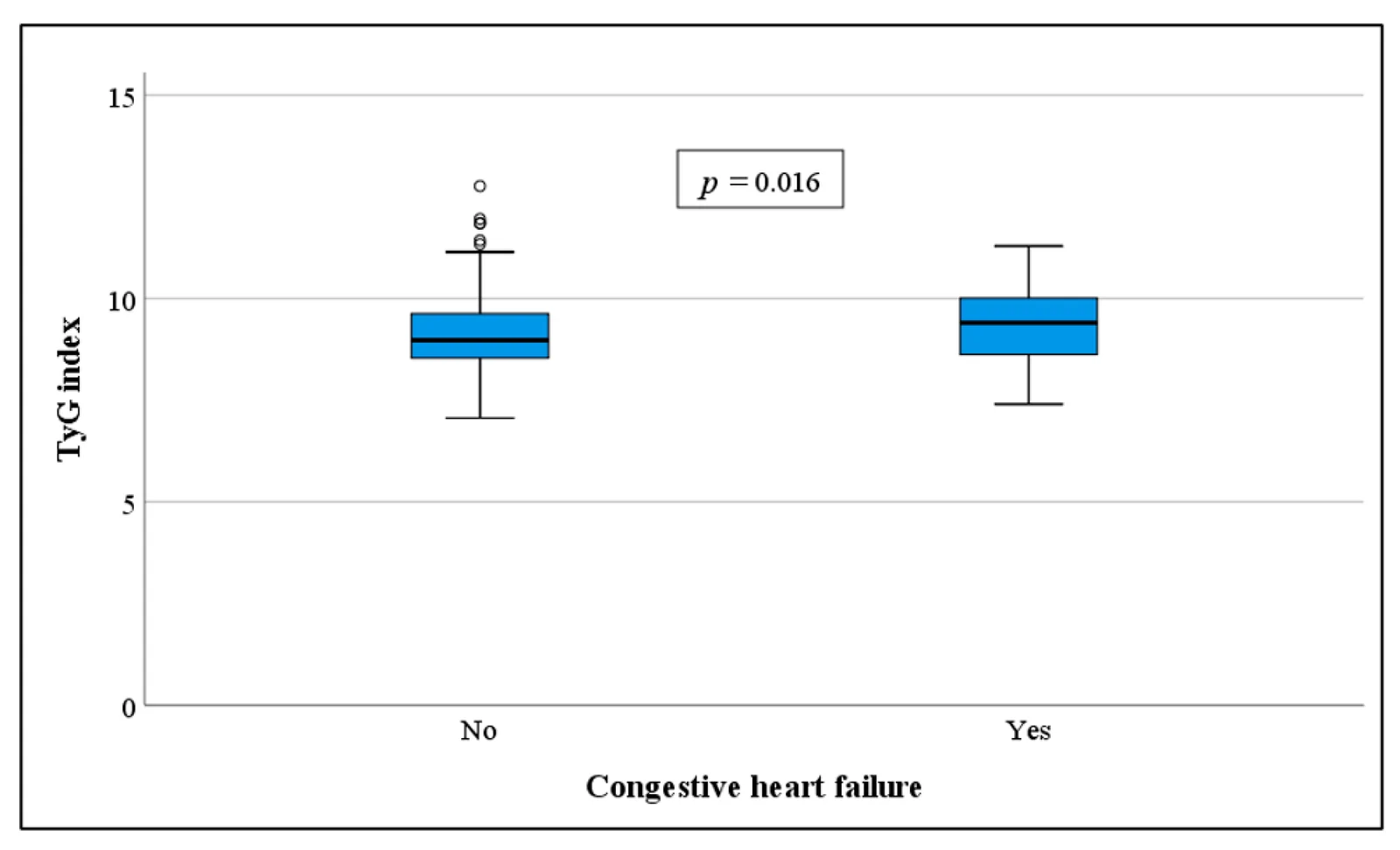
Comparison of TyG (triglyceride-glucose) index according to the presence of congestive heart failure.

Patients were further compared according to the development of congestive heart failure in terms of SHR and TyG index values, and both parameters were found to be significantly higher in patients with congestive heart failure (*p* = 0.002 and *p* = 0.016, respectively) (Figure 2 and Figure 3).

In addition, SHR index values were significantly higher in patients who patients who died during hospitalization compared to survivors [1.60 (1.42–2.22) vs. 1.07 (0.91–1.35), *p* < 0.001]. Similarly, TyG index values were significantly elevated in patients with mortality compared to those without mortality [9.75 (9.22–10.19) vs. 9.00 (8.54–9.64), *p* = 0.004].

**Figure 4:**
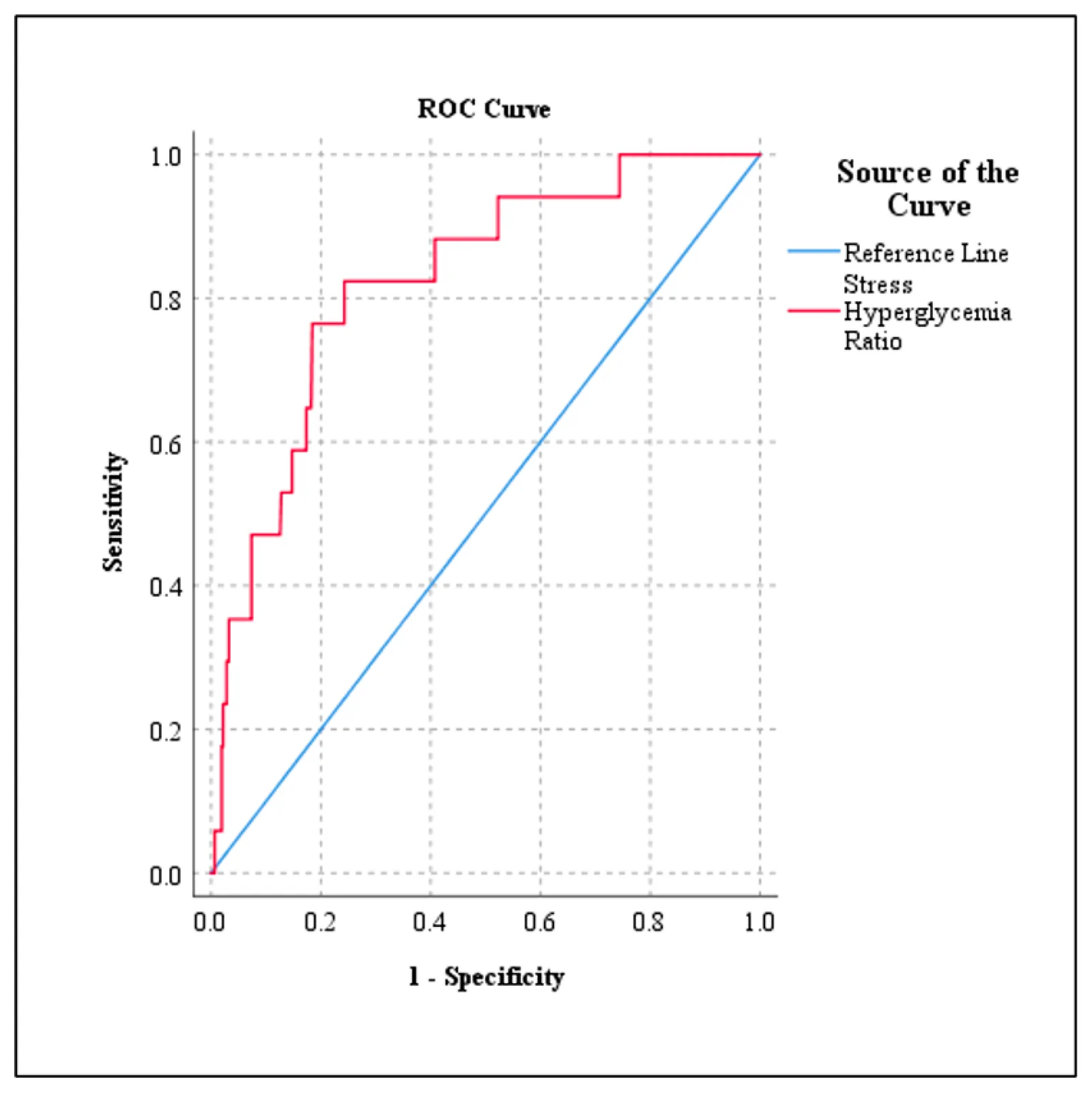
ROC curve analysis of the SHR index for predicting mortality.

The predictive performance of the SHR index for mortality was evaluated using ROC curve analysis and was found to be statistically significant [AUC: 0.823 (95% CI: 0.727–0.920), *p* < 0.001]. At a cut-off value of 1.45, the SHR index predicted mortality with 76.5% sensitivity and 80% specificity (Figure 4).

**Figure 5:**
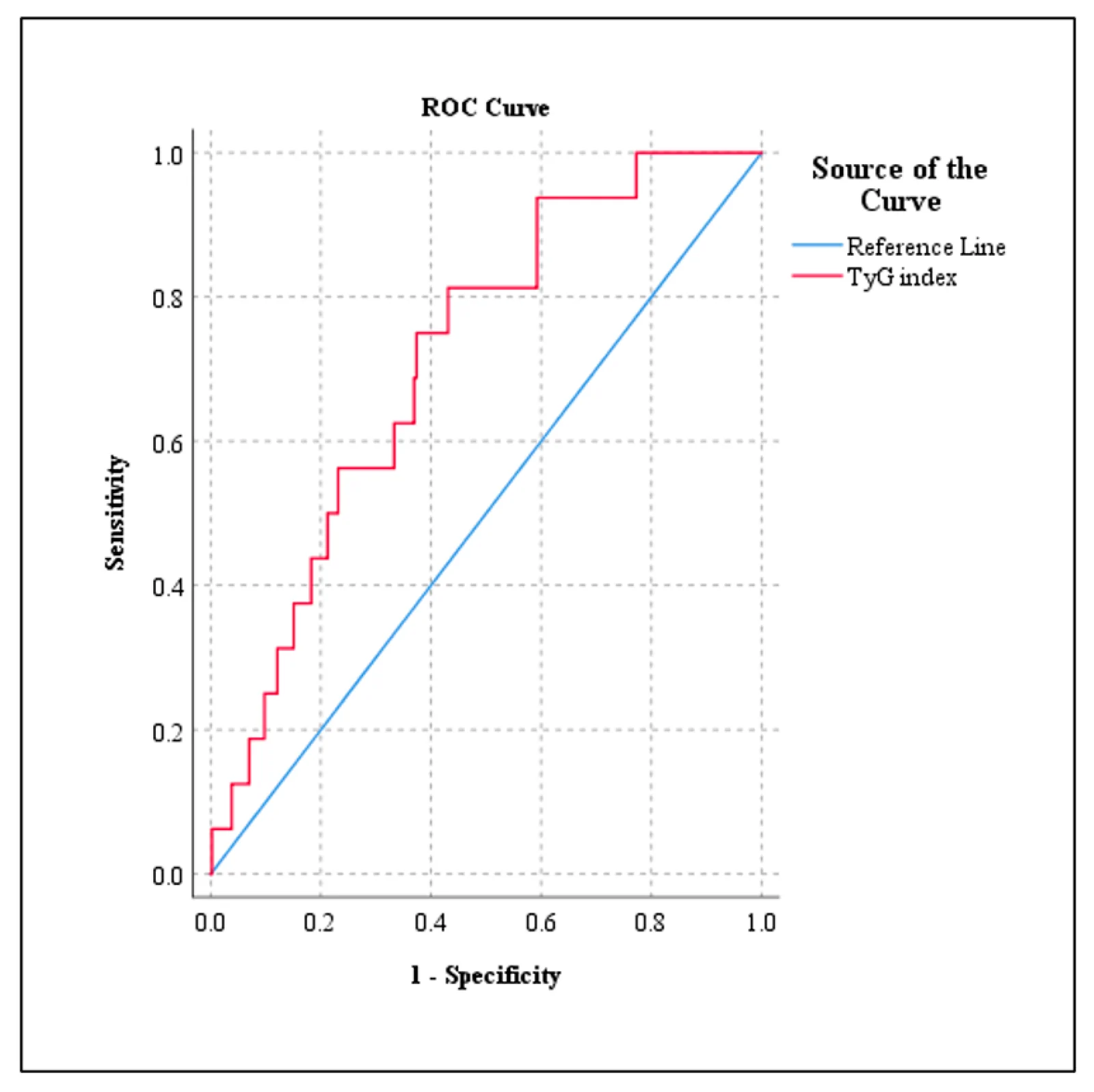
ROC curve analysis of the TyG (triglyceride-glucose) index for predicting mortality.

Similarly, the predictive performance of the TyG index for mortality was evaluated using ROC curve analysis [AUC: 0.714 (95% CI: 0.605–0.824), *p* < 0.001]. At a cut-off value of 9.41, the TyG index predicted mortality with 62.5% sensitivity and 66.7% specificity (Figure 5).

In-hospital mortality occurred in 17 of the 494 patients (3.4%).

Univariable logistic regression analysis demonstrated that SHR ≥ 1.45, TyG > 9.40, male sex, and diabetes mellitus were significantly associated with in-hospital mortality. Variables with *p* < 0.10 in univariable analysis were subsequently included in the multivariable logistic regression model.

In the multivariable analysis, SHR ≥ 1.45 remained associated with in-hospital mortality (OR: 9.61, 95% CI: 2.599–35.578; *p* = 0.001), whereas TyG > 9.40 was not statistically significant after adjustment (OR: 0.79, 95% CI: 0.215–2.899; *p* = 0.720).

## 4. Discussion

In the present study, we demonstrated that both the triglyceride–glucose (TyG) index and stress hyperglycemia ratio (SHR) were significantly associated with adverse in-hospital outcomes in patients presenting with acute myocardial infarction (AMI) and multivessel coronary artery disease. The principal findings of our study can be summarized as follows: (1) both TyG index and SHR levels were significantly higher in patients who developed congestive heart failure; (2) elevated SHR and TyG levels were associated with increased in-hospital mortality; (3) SHR and TyG indices demonstrated a significant positive correlation, suggesting an interaction between chronic insulin resistance and acute stress-related metabolic dysregulation; and (4) among the two indices, SHR emerged as an independent predictor of in-hospital mortality and demonstrated superior discriminative performance on ROC analysis.

A key finding of the present study was the significant positive correlation observed between SHR and the TyG index. Although these two parameters reflect different metabolic pathways, they appear to be closely interconnected in patients with AMI. While the TyG index primarily reflects chronic insulin resistance and long-term metabolic dysfunction, SHR represents acute stress-related glycemic dysregulation occurring during the acute phase of myocardial infarction. This relationship suggests that patients with underlying insulin resistance may be more vulnerable to exaggerated stress hyperglycemic responses during acute ischemic events, resulting in a compounded metabolic risk profile.

Several pathophysiological mechanisms may explain the association between acute hyperglycemia and adverse cardiovascular outcomes [9,10]. Acute stress hyperglycemia is associated with excessive catecholamine release, activation of inflammatory pathways, oxidative stress, endothelial dysfunction, platelet activation, and microvascular impairment. These processes may contribute to worsening myocardial injury, larger infarct size, impaired myocardial perfusion, and hemodynamic instability during AMI. Furthermore, acute hyperglycemia may aggravate myocardial oxygen demand and impair coronary microcirculatory function, thereby increasing the risk of heart failure and mortality [13,14].

Our results also demonstrated that SHR showed superior predictive performance for in-hospital mortality compared with the TyG index. More importantly, SHR remained an independent predictor of mortality after multivariable adjustment, whereas the prognostic significance of the TyG index was attenuated in multivariate analysis. Given the small number of mortality events, potential model overfitting, and incomplete adjustment for infarct severity and other clinical risk factors, these findings do not establish that acute glycemic stress has a greater prognostic impact than chronic insulin resistance. In the setting of acute ischemic injury, rapid neurohormonal and inflammatory activation may outweigh the contribution of chronic metabolic burden in predicting immediate adverse clinical outcomes. The loss of independent prognostic significance of the TyG index after multivariable adjustment may indicate that chronic metabolic dysfunction alone is insufficient to predict short-term mortality during acute ischemic stress.

Patients with multivessel coronary artery disease represent a particularly high-risk population characterized by diffuse atherosclerotic burden, endothelial dysfunction, and increased inflammatory activity [3,5,7]. In these patients, the combined effects of chronic insulin resistance and acute stress-related hyperglycemia may become more pronounced, potentially contributing to worse clinical outcomes. Therefore, assessment of metabolic indices reflecting both chronic and acute metabolic disturbances may provide incremental prognostic information in this vulnerable patient population.

Previous studies have demonstrated significant associations between elevated TyG index levels and adverse cardiovascular outcomes in patients with coronary artery disease and acute coronary syndromes [11,12,16]. Similarly, recent investigations evaluating SHR have reported strong associations between stress hyperglycemia and mortality, heart failure, and infarct severity in patients with AMI [8,13,15]. Our findings are generally consistent with the existing literature and further expand current evidence by simultaneously evaluating both chronic insulin resistance and acute stress-related hyperglycemia in a high-risk AMI population with multivessel coronary artery disease.

The clinical implications of our findings are noteworthy. Both the TyG index and SHR are inexpensive, easily obtainable, and reproducible biomarkers derived from routinely measured laboratory parameters. In particularly, SHR may serve as a practical tool for early identification of high-risk patients requiring closer hemodynamic monitoring and more intensive therapeutic strategies during hospitalization. Early recognition of high-risk patients using simple metabolic indices may also help optimize monitoring intensity, hemodynamic follow-up, and therapeutic decision making during the acute phase of myocardial infarction. In addition, the combined assessment of chronic metabolic dysfunction and acute stress-related hyperglycemia may provide a more comprehensive characterization of cardiometabolic risk in patients with AMI.

## 5. Study Limitations

The findings of this study should be interpreted in light of certain methodological limitations. First, the retrospective, single-center design may introduce selection bias and limit generalizability. The inclusion of patients who underwent coronary angiography and had complete laboratory data may have excluded individuals who died before these assessments, potentially underestimating in-hospital mortality. In addition, incomplete screening and exclusion records prevented the construction of a comprehensive patient selection flow diagram.

Second, the study included only patients with ST-segment elevation myocardial infarction (STEMI) undergoing primary percutaneous coronary intervention (PCI). Therefore, the findings may not be generalizable to patients with non-ST-segment elevation myocardial infarction (NSTEMI) or those receiving alternative treatment strategies.

Third, the limited number of in-hospital deaths (*n* = 17) relative to the number of predictors in the multivariable logistic regression model may have increased the risk of overfitting and reduced the precision of the estimated associations. The wide confidence interval for SHR and the absence of external validation further warrant cautious interpretation. Additionally, a formal statistical comparison of the ROC curves using the DeLong test was not performed; therefore, the statistical significance of the difference between the AUC values could not be established.

Furthermore, detailed information regarding the causes and timing of in-hospital deaths was unavailable in the retrospective analytical dataset, preventing cause-specific and temporal mortality analyses.

These considerations highlight the need for larger, prospective, multicenter studies with adequate numbers of clinical events and external validation to confirm the prognostic utility of SHR and the TyG index.

## 6. Conclusions

In conclusion, both the triglyceride–glucose (TyG) index and stress hyperglycemia ratio (SHR) were significantly associated with adverse in-hospital outcomes in patients with STEMI undergoing primary PCI.SHR showed a numerically higher AUC for in-hospital mortality than TyG, but a formal comparison of the ROC curves was not performed. The association between SHR and mortality in the original multivariable model should be interpreted cautiously because of the small number of deaths and potential residual confounding. These findings do not establish that acute glycemic stress outweighs chronic insulin resistance or that combining the indices improves prediction. The combined assessment of chronic insulin resistance and acute stress hyperglycemia may provide a practical approach to early risk stratification in high-risk patients with AMI. Given the limited number of in-hospital mortality events (*n* = 17), these findings should be considered exploratory and hypothesis-generating rather than definitive. Larger, prospective, multicenter studies with an adequate number of clinical events are needed to validate the prognostic utility of SHR and TyG in patients with STEMI undergoing primary PCI.

## Figures and Tables

**Table 1:** Baseline demographic and clinical characteristics stratified by in-hospital mortality status.

	Total (*n* = 494)	Survivors (*n* = 477)	Non-Survivors (*n* = 17)	*p* Value
**Age, years**	59.7 ± 12.1	59.4 ± 11.9	68.4 ± 13.2	0.020
**Gender, ** ***n*** ** (%) ** *Female* *Male*	167 (33.8) 327 (66.2)	155 (32.5) 322 (67.5)	12 (70.6) 5 (29.4)	0.030
**Chronic disease, ** ***n*** ** (%) ** *Hypertension* *Diabetes* *Previous myocardial infarction* *Cerebrovascular disease*	224 (45.3) 135 (27.3) 29 (5.9) 20 (4.0)	215 (45.1) 126 (26.4) 28 (5.9) 16 (3.4)	9 (52.9) 9 (52.9) 1 (5.9) 4 (23.5)	0.622 0.024 1.00 0.003
**Smoking, ** ***n*** ** (%)**	303 (61.3)	295 (61.8)	8 (47.1)	0.310

**Table 2:** Baseline laboratory characteristics stratified by in-hospital mortality status.

	Total (*n* = 494)	Survivors (*n* = 477)	Non-Survivors (*n* = 17)	*p* Value
**Glucose** (mg/dL)	138 (110–193)	136.5 (110–189)	197 (149.5–339)	<0.001
**HbA1C** (%)	5.9 (5.5–6.9)	5.9 (5.5–6.9)	6.2 (5.75–6.9)	0.320
**CRP** (mg/L)	4.0 (3.0–8.0)	4.0 (3.0–8.0)	14.0 (5.0–23.0)	<0.001
**HDL** (mg/dL)	40 (34–47)	40 (34–47)	38 (34–50)	0.890
**LDL** (mg/dL)	131 (112–159)	131 (112–159)	138 (114–177)	0.458
**TG** (mg/dL)	123 (76–191)	122 (74–190)	140 (97–231)	0.223
**Total cholesterol** (mg/dL)	196 (173–226)	195 (173–225)	196 (169–260)	0.622

**Abbreviations:**  CRP; C-reactive protein, HDL; high-density lipoprotein, LDL; low-density lipoprotein, TG; triglyceride. **Note**. Continuous variables were expressed as median (interquartile range).

## Data Availability

The data supporting the findings of this study are available from the corresponding author upon reasonable request and in accordance with applicable ethical and privacy restrictions.

## Abbreviations

The following abbreviations are used in this manuscript:

STEMI	ST-segment elevation myocardial infarction
PCI	percutaneous coronary intervention
AMI	Acute Myocardial Infarction
TyG	Triglyceride–Glucose
MVCAD	Multivessel Coronary Artery Disease
SHR	Stress Hyperglycemia Ratio
CHF	Congestive Heart Failure
CAD	Coronary Artery Disease
LDL-C	Low-Density Lipoprotein Cholesterol
HDL-C	High-Density Lipoprotein Cholesterol
CRP	C-Reactive Protein
HbA1c	Hemoglobin A1c
